# Impaired plasma phospholipids and relative amounts of essential polyunsaturated fatty acids in autistic patients from Saudi Arabia

**DOI:** 10.1186/1476-511X-10-63

**Published:** 2011-04-22

**Authors:** Afaf K El-Ansary, Abir G Ben Bacha, Layla Y Al- Ayahdi

**Affiliations:** 1 Biochemistry Department, Science College, King Saud University, P.O Box 22452, Zip code 11495, Riyadh, Saudi Arabia; 2 Autism Research and Treatment Center, King Saud University, P O Box 2925 Riyadh 11461 Saudi Arabia; 3 Shaik AL-Amodi Autism Research Chair, King Saud University, P O Box 2925 Riyadh 11461 Saudi Arabia; 4 Medicinal Chemistry Department, National Research Centre, P O Box 12622, Dokki, Cairo, Egypt; 5 Department of Physiology, Faculty of Medicine, King Saud University, P O Box 2925 Riyadh 11461 Saudi Arabia

**Keywords:** Autism, Polyunsaturated fatty acids, Essential fatty acids, Phospholipids, oxidative stress

## Abstract

**Backgrounds:**

Autism is a developmental disorder characterized by social and emotional deficits, language impairments and stereotyped behaviors that manifest in early postnatal life. This study aims to compare the relative concentrations of essential fatty acids (Linoleic and α- linolenic), their long chain polyunsaturated fatty acids and phospholipids in plasma of autistic patients from Saudi Arabia with age-matching controls.

**Methods:**

25 autistic children aged 3-15 years and 16 healthy children as control group were included in this study. Relative concentration of essential fatty acids/long chain polyunsaturated fatty acids and omega-3/omega-6 fatty acid series together with phosphatidylethanolamine, phosphatidylserine and phosphatidylcholine were measured in plasma of both groups.

**Results:**

Remarkable alteration of essential fatty acids/long chain polyunsaturated fatty acids, omeg-3/omega-6 and significant lower levels of phospholipids were reported. Reciever Operating characteristics (ROC) analysis of the measured parameters revealed a satisfactory level of sensitivity and specificity.

**Conclusion:**

Essential fatty acids/long chain polyunsaturated fatty acids and omeg-3/omega-6 ratios, phosphatidylethanolamine, phosphatidylserine and phosphatidylcholine could be used as potential biomarkers that point to specific mechanisms in the development of autism and may help tailor treatment or prevention strategies.

## Introduction

Autism is the most commonly studied of a spectrum of developmental disorders that are believed to be neurobiologically based but which, at this point, for lack of good biomarkers, are defined purely by behavior [1]. The number of cases has risen dramatically, and various hypotheses have been put forward to explain this phenomenon. Early estimates of the prevalence of this spectrum of disorders identified not less than 10 in 10,000 individuals as possessing some form of autism [2]. Autistic symptoms may also differ qualitatively from symptoms that characterize other disorders. Attention deficit, hyperactivity, and impulsivity are common in children with autism. Children with autism also have the ability to hyperfocus on activities of interest to them, such as spending hours twirling a string or reading a book.

Essential fatty acids (EFAs) taken in diets mediate brain functions and structures during development and are involved in many brain related disorder like autism. Fatty acids are commonly classified as saturated, monounsaturated, and polyunsaturated (PUFA) fatty acids, depending on the chemical structure and the length of the chain that can vary from 12 to 26 carbon bonds. Two types of PUFAs are EFA: linoleic acid (LA: 18:2, n-6) and α-linolenic acid (ALA: 18:3, n-3). The brain cannot distinguish among longer chain fatty acids that have been synthesized in the brain, and those that have been obtained from diet and crossed the blood-brain barrier. Clearly, the blood-brain barrier is a key to the bioavailability of brain EFA and PUFAs.

Among the significant components of cell membranes are the phospholipids that contain fatty acids. The brain phospholipids are exceptionally rich in PUFAs [3,4] and in contrast to other bodily tissue, a unique feature of neurons is the smaller amounts of the precursors LA and ALA and the higher amounts of their metabolites: arachidonic acid (AA: 20:4, n-6) and docasahexaenoic acid (DHA: 22:6, n-3) [3-6]. The two major PUFAs in all vertebrates are AA and DHA [7] and account for 20% of the dry brain weight [3,8]. DHA is known to be involved in cell signaling and cell proliferation [9] and has an important structural role in the brain [10], whilst AA is crucial for brain growth. Eicosapentaenoic (EPA: 20:5, n-3) seemingly has no structural role, but it is considered vital for the regulation of brain function [10]. LysoPC is a preferred carrier form of DHA to the brain [11]. On the other hand, there is evidence that entry of PUFAs into brain microvessels involves phospholipase A2 (PLA2) and lipoprotein- induced methylation of phosphatidylethanolamine (PE) [12].

Abnormalities in the fatty acid compositions of phospholipids, the major constituents of cell membranes, have been implicated in several neurodevelopmental disorders that manifest with psychiatric symptoms. For example, in schizophrenia, changes of red blood cell (RBC) membrane phospholipids such as deficiencies in n-3 PUFAs have been reported [13-16]. Indeed, supplementing diets with fish oil was shown to correct these deficiencies and lead to improvements in the symptom scores of schizophrenic patients [17]. Similarly, defects of fatty acids and phospholipids have recently been reported in autism subjects, including not only reduced levels of n-3 PUFAs, but also increased levels of saturated fatty acids in the RBC membrane [18] or in plasma [3, El-Ansary A, Ben Bacha A, Al-Ayadhi L: Plasma fatty acids as diagnostic markers in autistic patients from Saudi Arabia, Submitted]. Further evidence from Bell et al. (2004) [19] suggested that decreased levels of AA, docosatetraenoic acid (DTA) and DHA in RBC membranes from autism subjects could be caused by increased activity of RBC type IV PLA2, suggesting that altered metabolism of phospholipids may occur in autism [19].

This information initiates our interest to evaluate the plasma levels of phosphatidyl choline (PC), phosphatidylserine (PS) and PE as three important members of brain phospholipids in autistic patients. Moreover the relative amounts of EFA and PUSFA will be measured in a trial to highlight the possibility of using these lipid-related parameters as diagnostic markers.

## Materials and methods

### Reagents and chemicals

Chloroform and methanol used for extraction, ammonia (NH_3_) and water methanol for the mobile phase were 99% HPLC grade and obtained from Sigma-Aldrich (Taufkirchen, Germany). PC, PS and PE were obtained from Fluka, Sigma-Aldrich (Taufkirchen, Germany).

### Subjects and methods

The study protocol followed the ethical guidelines of the most recent Declaration of Helsinki (Edinburgh, 2000). All subjects enrolled in the study (25 autistics and 16 controls) had written informed consent provided by their parents and assented to participate if developmentally able. They were enrolled through the ART Center (Autism Research & Treatment Center) clinic. The ART Center clinic sample population consisted of children diagnosed on the autism spectrum (ASD). The diagnosis of ASD was confirmed in all subjects using the Autism Diagnostic Interview-Revised (ADI-R) and the Autism Diagnostic Observation Schedule (ADOS) and 3DI (Developmental, dimensional diagnostic interview). The ages of all autistic children participate between the ages of 4 and 12 years old. All were simplex cases. All are negative for fragile x gene study. The control group recruited from well baby clinic at king Khaled university hospital with mean age 4-11 years old. Subjects were excluded from the investigation if they had dysmorphic features, or diagnosis of Fragile X or other serious neurological (e.g., seizures), psychiatric (e.g., bipolar disorder) or known medical conditions. All participants were screened via parental interview for current and past physical illness. Children with known endocrine, cardiovascular, pulmonary, liver, kidney or other medical disease were excluded from the study.

### Samples collection

Blood samples were collected in the morning following at least 10 hour period of fasting. Plasma was collected using standard clinical practices and stored at -80°C until thawed for analysis.

### Lipid extraction and chromatography

- *Measurement of EFA and PUSFA: *Plasma (200 μl) lipids were extracted in the presence of internal standards and FA methylated using 3N methanolic HCL in sealed vials under nitrogen and incubated at 100C for 45 min. The methyl esters of free fatty acids were extracted with hexane, and the fatty acid composition of the extract was analyzed on a gas chromatograph (Helwlett-Packard 5890 series II plus, HP analytical Direct, Wilmington, DE), equipped with a flame ionization detector and a 30 m × 0.25 mm × 0.25 μm capillary column( Omegawax 250# 2-4136, Supelco). The helium gas flow rate was 1.2 ml/min, with a split/flow ratio of 50:1. Oven temperature was held at 205°C. The injector and detector temperatures were 260 and 262°C, respectively. Two internal standards, C15:0 and C23:0, were added during analysis. Fatty acids were identified via comparison of retention times with authentic standards [20].

- *Phospholipids Measurement: *Phospholipid separation was performed on a Kaneur Maxi Star HPLC system with four solvent lines, a degasser SEDEX 55 evaporating light detector (SEDEX 55 Lichtstreu detector, S.E.D.E.E., France) which was coupled with Apex M625 software (Autochrom, USA). As the nebulizing gaz, N_2 _was used at a flow rate of 4l/min, and a nebulizing temperature of 40°C. The gain was set at 8 and 2.0 bar N_2_.

A 125 × 4.0 mm Si-60 column with 5 μm particle diameter (Lichrosher) was used. The elution program was a linear gradient with 80:19.5:0.5 (V/V) chloroform: methanol: water: ammonia (NH_3_) at 22 min and the column was allowed to equilibrate until the next injection at 27 min. The injection volume was 50 μl. A liquid phase extraction procedure adapted from the method described by Bligh and Dyer (1959) was used to extract the serum samples.

Briefly, 50 μl of sample was diluted with 750 μl deionized water and mixed well. Then 2 ml of methanol and 1 ml of chloroform were added to the sample and mixed well. Then the mixture was homogenized (Rotary mixture 34526, Snijders) for 15 min. The mixture was centrifuged for 5 min by 4000 rpm.

### Statistical analysis

An SPSS computer program was used. Results were expressed as mean ± S.D. and all statistical comparisons were made by means of independent t-Test with P ≤ 0.005 was considered significant. Reciever Operating Characteristics analysis (ROC) was performed. Area under the curve, cutoff values together with degree of specificity and sensitivity were calculated. ROC curves are constructed by plotting the false positive rate (i.e. 100-specificity) against the true positive rate (i.e. sensitivity). These have been widely accepted as standard tools for evaluating the performance of diagnostic tests. The AUC is an overall summary of diagnostic accuracy, incorporating both components of accuracy, i.e., sensitivity and specificity, into a single measure. The AUC has been widely used as a quantitative index of the performance of a biomarker in a variety of applied fields; it is a simple and convenient overall measure of diagnostic test [21,22].

## Results and Discussion

EFAs and their long chain PUFAs are of critical importance in fetal growth and development [23-26]. These fatty acids are the precursors of eicosanoids and are essential constituents of the membrane lipids that maintain cellular and organelle integrity and important intracellular mediators of gene expression [27-29].

Table 1 shows that autistic patients of Saudi Arabia recorded a remarkable higher LA/AA and ALA/DHA ratios compared to age-matching controls. It appears that the same enzymes catalyze the conversion of both omega-6 and omega-3 fatty acid precursors into PUFAs. As reported by Pawlosky et al (2001) [30], the first step of the conversion process, ALA to EPA, is the rate-limiting step, with only 0.2% of ALA converted to EPA. The enzyme responsible for this conversion also converts docosapentaenoic acid (DPA) to the final product DHA but at a much higher rate (37%), suggesting that the enzyme has a low affinity for ALA. These enzymes are regulated by a negative feedback loop, suggesting that the dietary balance of different fatty acids is important [31]. The desaturation enzymes are only fully induced when levels of PUFAs are low, whereas if there is an adequate level of fatty acids, these desaturases are suppressed. Based on these information, we can suggest that a poorer ability to convert 18-carbon FA (LA and ALA) to their longer and more highly unsaturated derivatives could be considered in autistic patients of Saudi Arabia. This explanation could be supported through considering the previous work of Herault et al. (1993) [32] who detected a site linked to autism located on chromosome 11q22-23, in the viciny of the gene for delta-6 desaturase, which is the enzyme first involved in the production of PUFA-long chain derivatives of both (n-3) and (n-6) series [33]. The obtained results could find a support by the previous work of Richardson and Ross (2000) [16] in which an impairment of PUFAs metabolism has also been postulated to occur in children suffering from attention-deficit hyperactivity disorder (ADHD). This term is used to describe children, particularly boys, who are inattentive, impulsive and hyperactive [34]. Moreover, Mitchell et al. (1987) [35] and Stevens et al (1995) [36] showed that the proportions of DHA and AA were significantly lower in the plasma of ADHD children. They recorded that approximately 40% of subjects with ADHD had a greater frequency of symptoms indicative of EFAs deficiency (increased thirst, frequent urination, high fluid consumption, dry hair), compared to age- matching controls. The decreased levels of AA and DHA reported in the present study may be caused by increased activity of phospholipase A2 [19].

**Table 1:** Mean ± S.D of LA/AA, ALA/DHA, AA/DHA, EPA/DHA and EPA/AA ratios in plasma of autistic patients (N = 25) compared to age- matching controls (N = 16).

Parameters	Groups	**Min**.	**Max**.	Mean ± S.D	P value
**LA/AA**	Control	0.48	1.02	0.62 ± 0.16	0.034
		
	Autistic	0.41	3.31	1.08 ± 0.90	

**ALA/DHA**	Control	0.17	1.26	0.59 ± 0.36	0.004
		
	Autistic	0.43	1.18	0.84 ± 0.19	

**AA/DHA**	Control	0.47	0.99	0.81 ± 0.17	0.000
		
	Autistic	0.19	0.61	0.33 ± 0.14	

**EPA/DHA**	Control	0.17	1.44	0.56 ± 0.39	0.576
		
	Autistic	0.42	1.07	0.61 ± 0.19	

**EPA/AA**	Control	0.24	1.46	0.66 ± 0.37	0.000
		
	Autistic	1.01	3.49	0.96 ± 0.20	

Fatty acids are expressed in mmol/L plasma. Significant level at p < 0.01

Autism exhibits a marked gender bias with approximately four times more males diagnosed than females [37]. Although prepubertal boys and girls have similar testosterone levels (3.9 vs. 4.7 ng/dL) [38], prepubertal girls have 8-fold higher levels of estrogen than prepubertal boys (0.6 vs. 0.08 pg/mL [39]. Estrogen is neuroprotective against glutamate-induced neurotoxicity [40,41]. Further evidence regarding the protective characteristics of circulating estrogen levels comes from the work of Djouadi and colleagues (1998) [42]. Extier et al. (2009) [43] strongly suggest that male testosterone down- regulates while female estradiol up-regulates the synthesis of long chain n-3 PUFAs from ALA. This information could be helpful to suggest that deficiency of AA and DHA in Saudi autistic patients is greatly contributed in the etiology of the disorder.

Table 1 also demonstrates a much lower AA/DHA in autistic patients of Saudi Arabia compared to age-matched control. This could be attributed to a combination of factors including altered enzyme activity affecting conversion of LA and ALA as precursor fatty acids and/or excessive utilization of these metabolites. The recorded lower AA/DHA reported in the present study in autistic patient could be also explained on the basis that within body tissues, DHA status fluctuates quite readily with dietary changes while AA concentrations remain relatively stable and that there is little difference in AA content amongst individuals [44]. Therefore, the occurrence of a subnormal AA concentration in Saudi autistics [El-Ansary A, Ben Bacha A, Al-Ayadhi L: Plasma fatty acids as diagnostic markers in autistic patients from Saudi Arabia, Submitted.] may be of particular importance than DHA and could be physiologically relevant to abnormal brain function. Table 1 also showed that autistic patients had a significantly higher EPA/AA ratio which confirmed that they have much higher n-3/n-6 fatty acids. This is in good agreement with the previous reports of Wiest et al. (2009) [45] that individuals with autism had higher concentrations of long-chain n-3 fatty acids in both PE and PC, and tended to have lower concentrations of long-chain n-6 fatty acids. They attributed this elevated ratio to the fact that plasma lipid profiles are known to be affected by increased consumption of n-3-rich food e.g fish, raising EPA and DHA.

Consistent with the increased oxidative stress biomarkers, Saudi children with autism were found to have increased body burdens of lipid peroxides [46]. A second line of evidence that oxidative stress may play a role in autism in Saudi population is suggested by a reduced endogenous antioxidant capacity. Specifically, altered glutathione peroxidase (GPX), superoxide dismutase (SOD), catalase activities as well as total GSH and vitamin E [46]. Lower AA concentration recorded in the present study could be easily related to the oxidative stress as an accepted mechanism in the etiology of autism. This suggestion could be supported through considering the previous work of Ming et al. (2005) [47] in which they recorded urinary excretion of 8-isoprostane (8-iso-PGF2a) a class of autoxidation products generated from AA acid by a free radical initiated process, in children with autism compared to age-matched controls.

In eukaryotic cells, phospholipids are the predominant membrane lipids and are, from a topographic point of view, asymmetrically distributed across the bilayer [48,49]. PC, PE, PS, are the major phospholipids [50]. In most eukaryotic membranes, PC and PE represent together around 60-85% of the phospholipids fraction, while for the other phospholipids small but significant differences can be found depending of the cell membrane and even animal species [51,52]. Phospholipids play multiple roles. They constitute a permeability barrier, modulate the functional properties of membrane-associated activities, provide a matrix for the assembly and function of a wide variety of catalytic processes, and act as donors during the synthesis of macromolecules. The wide range of processes in which phospholipids are specifically involved explains the need for diversity in phospholipid structures and fatty acid composition [53].

**Figure 1:**
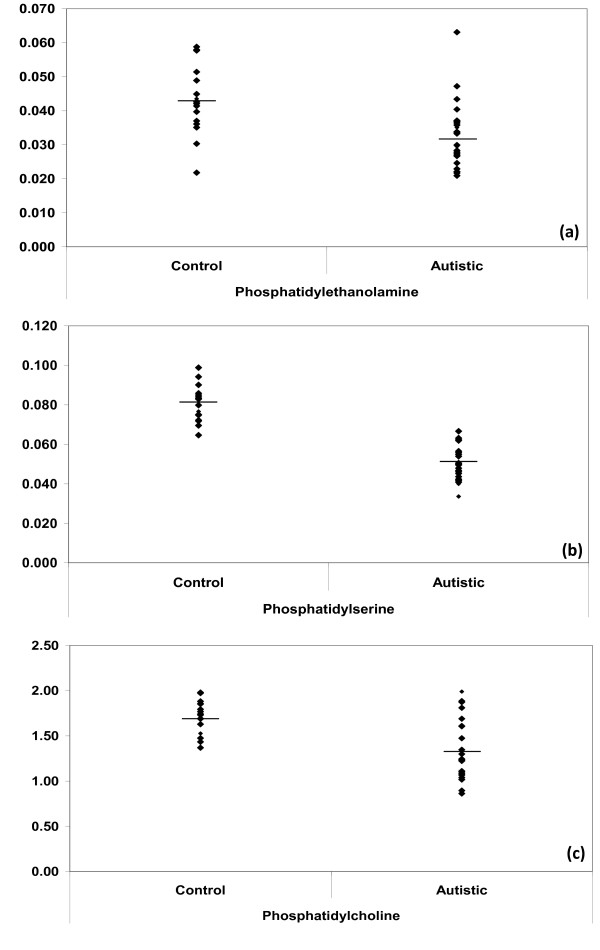
**Mean of measured phospholipids in autistic (N = 25) compared to age- matching controls (N = 16)**. Mean value for each group is designated by a line. **(a) **PE; **(b) **PS and **(c) **PC.

Table 2 and Figure 1 demonstrated that PE, PS and PC were significantly lower in autistic patients compared to healthy control. This could be easily correlated to certain autistic features specially those related to oxidative stress and inflammatory responses as two mechanisms have been shown to play a critical role in the pathophysiology of autism. This could find a support through considering the reports of Pandey et al. (2009) [54] that Omega-6 phospholipids, e.g. PC have been shown to have anti-inflammatory properties through inhibiting tumor necrosis factor (TNF-α) and H_2_O_2 _activated mitogen-activated protein kinase (MAPK) in neuronal cell line SH-SY5Y cells and prevents the phosphorylation and activation of nuclear factor-kappa B. The lower concentration of PC reported in the present study could explain the previously proved H_2_O_2 _intoxication in plasma samples collected from the same autistic patients [46] and explain the increase of pro-inflammatory cytokines in autistic patients [55].

**Table 2:** Mean ± S.D of plasma levels of PC, PS and PC (expressed in mmol/L plasma) in autistic patients compared to age- matching controls.

Parameter	Group	N	Minimum	Maximum	Mean ± S.D	P value
**PE**	Control	16	0.022	0.059	0.043 ± 0.010	0.002
		
	Autistic	25	0.021	0.063	0.032 ± 0.009	

**PS**	Control	16	0.065	0.099	0.081 ± 0.009	0.000
		
	Autistic	25	0.034	0.067	0.051 ± 0.009	

**PC**	Control	16	1.369	1.979	1.711 ± 0.185	0.000
		
	Autistic	25	0.862	1.989	1.313 ± 0.333	

Significant level at p < 0.005

The obtained lower PS presented in Table 2 and Figure 1 in plasma of autistic patients compared to age- matching controls could be easily correlated to the over expression of superoxide dismutase (SOD) as a phenotype previously reported by Al-Gadani et al. (2009) [46] in autistic patients from Saudi Arabia. Glozman et al. (2000) [56] proved that Cu/Zn-SOD gene are compatible with DHA and PS deficiency in the fetal, but not the adult brain.

It is well documented that phospholipids enriched in unsaturated fatty acids (PE, PS and PC) are crucial to the normal neurological function of the brain. Neurodegeneration has been shown to be associated with abnormal phospholipids metabolism in the brain [57]. Erythrocyte membrane phospholipid composition has been shown to correlate to brain phospholipid composition [58] and may be a useful marker for neurological disease [59,60].

**Figure 2:**
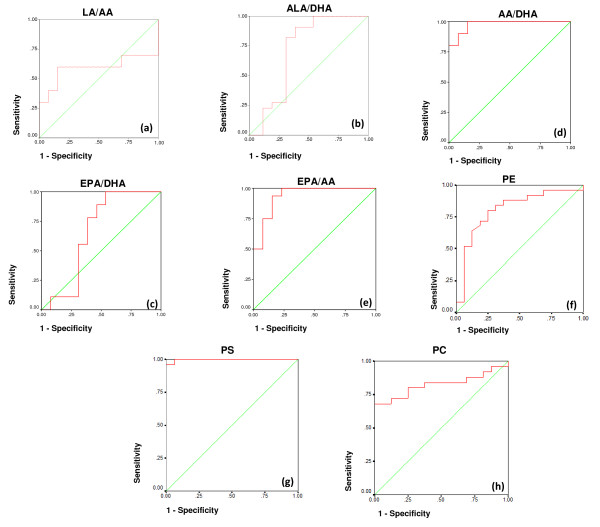
**ROC curves showing area under the curves, specificity and sensitivity of LA/AA (a), ALA/DHA (b), AA/DHA (c), EPA/DHA (d) and EPA/AA (e), PE (f), PS (g) and PC (h) in autistic patients (N = 25)**.

ROC analysis presented in table 3 together with figure 2 demonstrate that AA/DHA, EPA/AA and the three measured phospholipids (PE, PS and PC) showed a satisfactory specificity and sensitivity and they could be uses as biomarkers for the early diagnosis of autism in Saudi populations.

**Table 3:** ROC analysis of LA/AA, ALA/DHA, AA/DHA, EPA/DHA and EPA/AA ratios and PE, PS and PC in autistic groups (N = 25).

Parameter	Area under the curve	Cutoff value	Sensitivity %	Specificity %
**LA/AA**	0.592	0.69	60.0%	84.6%

**ALA/DHA**	0.713	0.57	90.9%	61.5%

**AA/DHA**	0.977	0.66	100.0%	84.6%

**EPA/DHA**	0.658	0.39	100.0%	46.2%

**EPA/AA**	0.937	1.07	93.8%	84.6%

**PE**	0.806	0.037	80.0%	75.0%

**PS**	0.998	0.068	100.0%	93.7%

**PC**	0.825	1.619	80.0%	75.0%

**Figure 3:**
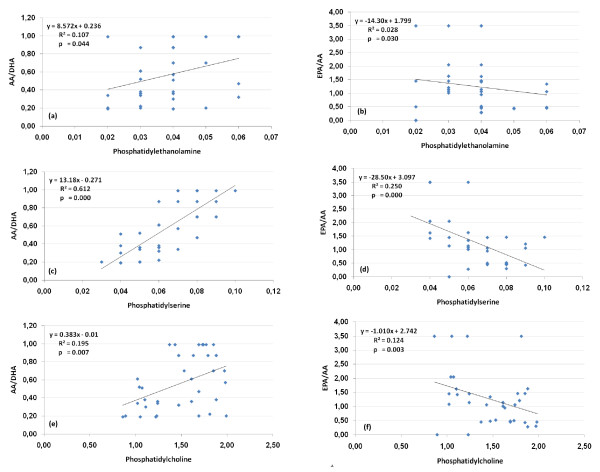
**Correlation between the measured phospholipids and fatty acids ratios for autistic patients (N = 25) with best fit line curve**: **(a) **PE and AA/DHA (positive correlation); **(b)**: PE and EPA/AA (negative correlation); **(c)**: PS and AA/DHA (positive correlation); **(d)**: PS and EPA/AA (negative correlation); **(e)**: PC and AA/DHA (positive correlation); **(f)**: PC and EPA/AA (negative correlation).

Figure 3 demonstrate the Pearson correlation test between fatty acid ratios and phospholipids concentrations. PE, PS and PC are correlated negatively with EPA/AA and positively with the AA/DHA which highlight the relationship between AA deficiency and the etiopathology of autism. This support the previously mentioned phenomena that subnormal concentration of AA is more related to autism than DHA and EPA as two PUSFAs greatly affected by diet.

In conclusion, since erythrocyte phospholipid composition is impacted by dietary habits, the therapeutic early administration of omega-6 phospholipids, such as PC, would be expected to increase the concentration of these lipids in both the plasma and brain and promote neuronal anti-inflammatory events.

## Abbreviations

**PUFA**: polyunsaturated fatty acid; **EFA**: Essential fatty acids; **LA**: linoleic acid; **ALA**: α-linolenic acid; **AA**: Arachidonic acid; **DHA**: Docasahexaneoic acid; **EPA**: Eicosapentaenoic; **PC**: phosphatidylcholine; **PE**: phosphatidylethanolamine; **PS**: phosphatidylserine; **ADHD**: attention-deficit hyperactivity disorder.

## Competing interests

The authors declare that they have no competing interests.

## Authors' contributions

AE: Designed the study and drafted the manuscript.

ABB: Helped to draft the manuscript and performed the statistical analysis.

LA: Provided samples and participated in the design of the study.

All authors read and approved the final manuscript.
